# The Role of Supplemental Complex Dietary Carbohydrates and Gut Microbiota in Promoting Cardiometabolic and Immunological Health in Obesity: Lessons from Healthy Non-Obese Individuals

**DOI:** 10.3389/fnut.2017.00034

**Published:** 2017-07-24

**Authors:** Petra C. Vinke, Sahar El Aidy, Gertjan van Dijk

**Affiliations:** ^1^Department of Behavioral Neuroscience, Groningen Institute for Evolutionary Life Sciences (GELIFES) – Neurobiology, University of Groningen, Groningen, Netherlands; ^2^Microbial Physiology, Groningen Biomolecular Sciences and Biotechnology Institute (GBB), University of Groningen, Groningen, Netherlands

**Keywords:** gut microbiota, prebiotics, obesity, cardiometabolic, inflammation, metabolic syndrome

## Abstract

Dietary supplementation with complex carbohydrates is known to alter the composition of gut microbiota, and optimal implementation of the use of these so called “prebiotics” could be of great potential in prevention and possibly treatment of obesity and associated cardiometabolic and inflammatory diseases *via* changes in the gut microbiota. An alternative to this “microbiocentric view” is the idea that health-promoting effects of certain complex carbohydrates reside in the host, and could secondarily affect the diversity and abundance of gut microbiota. To circumvent this potential interpretational problem, we aimed at providing an overview about whether and how dietary supplementation of different complex carbohydrates changes the gut microbiome in healthy non-obese individuals. We then reviewed whether the reported changes in gut bacterial members found to be established by complex carbohydrates would benefit or harm the cardiometabolic and immunological health of the host taking into account the alterations in the microbiome composition and abundance known to be associated with obesity and its associated disorders. By combining these research areas, we aimed to give a better insight into the potential of (foods containing) complex carbohydrates in the treatment and prevention of above-mentioned diseases. We conclude that supplemental complex carbohydrates that increase Bifidobacteria and Lactobacilli, without increasing the deleterious *Bacteroides*, are most likely promoting cardiometabolic and immunological health in obese subjects. Because certain complex carbohydrates also affect the host’s immunity directly, it is likely that host–microbiome interactions in determination of health and disease characteristics are indeed bidirectional. Overall, this review article shows that whereas it is relatively clear in which direction supplemental fermentable carbohydrates can alter the gut microbiome, the relevance of these changes regarding health remains controversial. Future research should take into account the different causes of obesity and its adverse health conditions, which in turn have drastic effects on the microbiome balance.

## Introduction

It is now well recognized that the bacteria living in our gut play an important role in the development and maintenance of the innate and adaptive immune system (1, 2) as well as the fermentation of low-, or non-digestible dietary carbohydrates (3–5). While the human intestinal microbiome is dominated by the phyla Bacteroidetes and Firmicutes (representing more than 90% of the total bacterial population) and with Proteobacteria, Verrucomicrobia, Actinobacteria, Fusobacteria, and Cyanobacteria generally being present in minor proportions (6), the exact composition can vary widely between individuals. Contributing factors to this variation are (1) the host’s genetic make-up (7), (2) early-life environmental conditions under which the host was born and nourished (i.e., cesarean section versus vaginal delivery, breast milk versus infant formula feeding, perinatal stress) (8–11), (3) the type and intensity of antibiotic treatment the host has undergone (12), and (4) the microbial composition that appears to change slowly with age (13–15). Whereas the acquired microbial abundance and diversity was initially thought to be relatively stable and withstand sudden perturbations (16, 17), it was found more recently that changes in the diet can affect microbial composition *after* its initial establishment (18–20). For diet-driven alterations of gut microbial composition particularly the type and proportion of complex indigestible carbohydrates that reach the colon turn out to be of great importance (18, 19, 21–24). The digestibility of dietary carbohydrates depends, among others, on structural properties of the carbohydrate, such as the degree of polymerization (DP) and branching. In general, carbohydrates with a low DP can rapidly be absorbed or digested in the small intestine. The larger oligosaccharides and polysaccharides have a variety of branching and polymerizations, which render them more difficult for host enzymes in the small intestine to be hydrolyzed (25). Carbohydrates with higher DP’s will, therefore, enter the large bowel, where they will be fermented by bacteria, which are highest in abundance in this part of the gastrointestinal tract (3, 4). Another factor for digestibility of polysaccharides is of course whether or not the digestive enzymes for specific cleavages are present in the digestive tract of an animal. For example, humans do not have the enzymes required to cleave the relevant linkages in, e.g., cellulose and beta-glucan, which render them indigestible and therefore reach the bowel where they can be fermented.

Besides playing a role in fermentation and immune functioning, the diversity and abundance of gut microbiota also plays a role in regulation of body weight and energy balance (26–28). This notion started with the discovery of Turnbaugh and colleagues in 2006 showing that transplantation of gut microbiota of genetically obese *ob/ob* mice into the gut of germ-free mice resulted in greater weight gain of the receiving animals than animals that received the microbiota of their lean counterparts (29). Comparable studies have since then been done, all adding to the conclusion that obesity risk, as well as related cardiometabolic and inflammatory derangements may rely, at least in part, on gut microbiota (30–34). Because of the apparent fact that the relative abundance and diversity of gut microbiota is related to the host’s health and/or disease status (20, 35), the possibility to affect the composition of intestinal microbiota by complex indigestible carbohydrates—so-called “prebiotics”—and subsequently improve the health of the host (36) has gained a lot of interest over the past few years.

Several randomized-controlled studies have been conducted over the years to elucidate the effects of supplementation of indigestible carbohydrates on several health indices in (1) healthy volunteers (37, 38), (2) subjects diagnosed with metabolic syndrome (MetS), type-2 diabetes mellitus (T2DM), cardiovascular disease (CVD), and/or inflammatory bowel disease (IBD), and (3) subjects at risk of having these diseases because of the fact that they are overweight or obese (39–43). It is difficult to evaluate the role of the microbiome in the reported health effect of supplemental carbohydrates. It was for example recently shown that a polymer of fructose molecules (i.e., β2→1-fructan) has a direct modulatory effects on immunity, which in turn affects the colonizing microbiota. Of note, the effects of the tested carbohydrates were dependent on the chain length of the β2→1-fructans type polymer (44). These results suggest that health-promoting effects of certain complex carbohydrates are actually residing in the host, and could secondarily affect the diversity and abundance of gut microbiota, thereby providing an alternative to the current “microbiocentric” view on health and disease. For this reason, we aimed at providing an overview, based on human trials, about whether and how dietary supplementation of different (potentially prebiotic) carbohydrates changes the gut microbiome in healthy individuals. We then reviewed whether the reported changes in gut bacterial members found to be established by prebiotic carbohydrates would benefit or harm the host’s health taking into account the alterations in the microbiome composition and abundance known to be associated with obesity, CVD, T2DM, and/or IBD. By combining these research areas, we aimed to give a better insight into the potential of (foods containing) prebiotic carbohydrates in the treatment and prevention of above-mentioned diseases.

## How Can Prebiotics Affect the Host Health through Gut Microbiota

Before discussing the studies investigating the health effects of supplemental carbohydrate on the microbiome, we first discuss a few potential mechanisms how these health effects can be substantiated. Microbial fermentation can convert the carbohydrate residues in, among others, short-chain fatty acids (SCFAs). These SCFAs comprise acetate, propionate, and butyrate (5) and can account for up to 10% of the energy absorbed from the diet, benefiting the microbiota as well as the host. The SCFAs acetate and propionate are mainly produced by Bacteroidetes, and butyrate is the primary product of the Firmicutes bacteria (45, 46). Besides the delivery of extra fuels from fermentation of complex carbohydrates that escape digestion, the host also benefits from these microbial fuels in other ways. For example, through their effects on gastric motility and intestinal transit stimulation, SCFAs stimulate serotonin release as reported in an *in vitro* colonic mucosal system (47). Locally butyrate functions as an important energy source for colonocytes, and this interaction contributes to a reduction in the risk of colorectal cancer (4, 48). Butyrate also affects fuel fluxes and energy balance by stimulating leptin and glucagon-like peptide 1 amide (49). Butyrate has even been reported to elicit antidepressant effects in the mouse (50) and to stimulate social behavior and facilitating long-term memory consolidation and neuroprotection/regeneration (51, 52). Importantly, SCFAs appear to also reduce the risk of CVD, IBD, T2DM, and diet-induced obesity (4). The processes underlying the aforementioned effects of SCFAs are subject to intense investigation, and several interesting leads have been elucidated.

Besides fermenting poorly or indigestible carbohydrates, resulting in among others increased levels of SCFAs, the gut microbiota can also modulate the uptake and deposition of dietary lipids by affecting the activity of the lipoprotein lipase in adipose tissues, an enzyme that promotes fatty acid uptake into adipocytes (33), and by affecting the synthesis of colipases, which are involved in the hydrolysis of dietary lipids (53). Endocrine (cortisol, gut hormones), immune (cytokines), and neural (vagus and enteric nervous system) routes have been suggested to play a role in the communication between gut microbiota (e.g., *via* the metabolites it produces upon digestion of prebiotics) and peripheral organs and the brain (54–57). Although underlying mechanisms are far from understood, it is likely that the communication between the central nervous system and gut microbiota functions bidirectionally (54, 57–59).

## Assessing Effects of Prebiotics in Healthy Subjects and Relevance for Those with Cardiometabolic/Inflammatory Diseases and Obesity

Food ingredients with prebiotic properties are often oligosaccharides and polysaccharides (Table 1) (25, 60–63), such as inulins, pectins, and β-glucan compounds. Most prebiotics are already included in the diet as they are naturally present in several types of food [e.g., legumes, wheat, rye, and onions (64, 65)], and are, therefore, considered to be part of a diet that contributes to the maintenance of sustainable health (66–68). Of note, although usually ignored in research reports, the (excessive) use of prebiotics could eventually lead to undesirable effects like excessive flatus, borborygmi, abdominal pain, and diarrhea (69, 70). To assess the effect of dietary supplemental (potentially prebiotic) carbohydrates on gut microbiota composition, a literature search was performed in PubMed and Google Scholar, as shown in Table 2. The initial literature search was performed in January 2016 and was revised in January 2017. The search term always included “gut microbiota OR microbiota,” which was then combined with the terms “composition,” “prebiotic,” and “polysaccharides” or oligosaccharides. Search results were then screened for clinical trials in healthy humans, which eventually narrowed down the results to 20 clinical studies. All studies included daily supplementation of (potentially prebiotic) carbohydrates for a minimum of 2 weeks and for a maximum of 10 weeks. We first subdivided these trails on the basis of the type of supplemental carbohydrates that were used in the different trials, and we then assessed the direction of change in the different bacterial phyla and/or genera. All studies used freshly voided fecal samples, and subsequently, a variety of methodologies were used to determine microbiota composition, including Florescent *in situ* hybridization (FISH), quantitative (q)PCR, 16S ribosomal RNA sequencing, and whole-genome shotgun sequencing.

**Table 1:** Overview of the major dietary carbohydrates.

Class (DP)	Subgroup	Principal components	Physiology	Possible source
Sugars (1–2)	Monosaccharides	Glucose, fructose, galactose	Absorbed	Fruits
	Disaccharides	Sucrose, maltose, trehalose	Absorbed	Sugar cane/beets
		Lactose	Absorbed/fermented	Dairy
	Polyols (sugar alcohols)	Sorbitol, mannitol, lactitol, xylitol, erythritol, isomalt, maltitol	Absorbed/fermented	Synthetic

Oligosaccharides (3–9) (short-chain carbohydrates)	Malto-oligosaccharides (α-glucans)	Maltodextrins	Digested/fermented	Glucose syrups
	Non-α-glucan oligosaccharides	Raffinose, stachyose	Fermented	Beans, peas, soya
		Fructo-oligosaccharides, galacto-oligosaccharides		Enzymatic synthesis
		Polydextrose		Synthetic
		Inulin		Wheat, onion, banana

Polysaccharides (≥10)	Starch (α-glucans)	Amylose, amylopectin, modified starches	Digested/fermented	Potatoes
	Non-starch polysaccharides	Cellulose, hemicellulose, pectin, arabinoxylans, β-glucan, glucomannans, plant gums and mucilages, hydrocolloids, soluble corn fiber	Fermented	Cell wall plant cells (fruits/vegetables)

*DP, degree of polymerization or number of monomeric (single sugar) units*.

*Table based on Cummings and Stephan (25), Cummings et al. (62), and Roberfroid (63)*.

**Table 2:** Overview of search terms and the number of hits in PubMed.

Search term	Total	Clinical trials
((gut microbiota) OR microbiota) AND carbohydrate	2,465	187
((gut microbiota) OR microbiota) AND prebiotic	1,077	106
((gut microbiota) OR microbiota) AND composition AND prebiotic	391	43
((gut microbiota) OR microbiota) AND composition AND prebiotic AND polysaccharides	232	36
((gut microbiota) OR microbiota) AND composition AND prebiotic AND oligosaccharides	106	18
((gut microbiota) OR microbiota) AND composition AND prebiotic AND polysaccharides AND human	190	35
((gut microbiota) OR microbiota) AND composition AND prebiotic AND oligosaccharides AND human	79	18

*Search terms were combined to find studies assessing the effect of potentially prebiotic carbohydrates on gut microbiota composition. Studies referred to in review articles published on the topic were also included in some cases. Only studies supplementing the diet of healthy individuals were included. Second, the microbiota genera that were found to be altered upon carbohydrate supplementation were then further investigated in a second literature search in which the name of the genera was combined with the search terms “correlation,” “health,” “obesity,” “diabetes,” “inflammation,” “cardio(-) metabolic disease,” and “cardio(-)vascular disease.” Again, studies referred to in review articles published on the topic were also included in some cases. Here, animal studies were included, in addition to human studies, because they can often be instrumental in explaining the health effects of the genera in the (sometimes scarce) human studies*.

Traditionally, the composition of the microbiota was analyzed by using culturing techniques, which select for specific bacterial populations based on their metabolic requirements. These techniques, however, are clearly only applicable to the culturable fraction of the members (i.e., which is only true for about 20–50%) and has limited resolution (6, 71, 72). Culture-independent techniques that are frequently used nowadays include 16S ribosomal RNA-based techniques such as qPCR and illumina (deep) sequencing. Last, whole-genome shotgun sequencing does not only sequence a marker gene such as the 16S ribosomal RNA but also examines the whole-genome in which it provides more detailed information about the functional and metabolic potential of the community (71, 72). An overview of the trials that were found by using the (combined) search terms (Table 2) is presented in Table 3 (24, 64, 73–91). We subsequently characterized the effects of the different supplemental carbohydrates per phylum, and for each of them discussed the feasibility of treating subjects diagnosed with obesity, cardiometabolic, and/or inflammatory diseases with any of these prebiotics.

**Table 3:** Overview of the studies in which the diet of healthy subjects was supplemented with prebiotic carbohydrates.

Carbohydrate group	Supplement	Treatment	Affected microbiota	−/+	*N*^a^	Analysis	Reference
Disaccharide	Maltitol	45.6 g/day—2 weeks	*Bifidobacterium*	+	40	Florescent *in situ* hybridization (FISH)	Beards et al. (73)
			*Bacteroides*	+			
			Lactobacilli	+			
			Eubacteria	+			
			*Atopobium*	+			

Oligosaccharide	Fermented soybean milk (containing raffinose/stachytose)	11.7 g raffinose + 53.5 g stachyose/day—2 weeks	*Bifidobacterium*	+	10	Plate count technique	Inoguchi et al. (74)
			Lactobacilli	+			
			Clostridia	−			
	
	Xylo-oligosaccharide	1.4 g/2.8 g/day—8 weeks	*Bifidobacterium*	+	32	bTE-FAP/16S rRNA gene sequencing	Finegold et al. (64)
	
	Xylo-oligosaccharide	2.8 g/day—8 weeks	*Bacteroides fragilis*	+	32	bTE-FAP/16S rRNA gene sequencing	Finegold et al. (64)
	
	Stachyose-enriched α-galacto-oligosaccharides	5 g/day—2 weeks	Bifidobacteria	+	50	Plate count technique	Li et al. (91)
			Lactobacilli	+			
			*Clostridium perfringens*	−			
	
	Galacto-oligosaccharide mixture: B-GOS	5.5 g/day—10 weeks	*Bacteroides*	+	45	FISH	Vulevic et al. (75)
			*Bifidobacterium*	+			
	
	Arabinoxylan-oligosaccharides	2.2 g/day—3 weeks	Lactobacilli	+	44	FISH	Walton et al. (76)
			*Bacteroides*	+			
	
	Arabinoxylan-oligosaccharides	10 g/day—3 weeks	*Bifidobacterium*	+	20	qRT-PCR	Cloetens et al. (77)
	
	Galacto-oligosaccharides	8 g/day—3 weeks	*Bifidobacterium*	+	39	qPCR	Walton et al. (78)
	
	Xylooligosachharide	5 g/day—4 weeks	*Bifidobacterium*	+	60	qPCR	Lecerf et al. (79)
	
	Xylo-oligosaccharide	2 g/day—8 weeks	*Catenibacterium*	−	7	qPCR	Yang et al. (90)
			*Blautia*	−			
			*Eubacterium*	−			
			*Subdoligranulum*	−			
			*Collinsella*	−			
			*Streptococcus*	−			
	
	Short-chain fructo-oligosaccharides + *Sideritis euboea* extract	5 g/day—30 days	*Bifidobacterium*	+	52	Plate count technique	Mitsou et al. (80)
	
	Resistent maltodextrin	50 g/day—24 days	*Bifidobacterium*	+	14	16S rRNA approach (DGGE-based profiling, qPCR, FISH, 454-titanium tech.-based 16S rRNA sequencing)	Baer et al. (81)

Polysaccharide	Resistant starch type 2	33 g/day—3 weeks	*Eubacterium rectale*	+	13	Selective culture, PCR-DGGE, qRT-PCR	Martínez et al. (82)
			*Rumminococcus bromii*	+			
	
	Resistant starch type 4	33 g/day—3 weeks	Actinobacteria (*Bifidobacterium*)	+	13	Selective culture, PCR-DGGE, qRT-PCR	Martínez et al. (82)
			Bacteroidetes (*Parabacteroides*)	+			
			Firmicutes (Ruminococcaceae)	−			
	
	Resistant starch + maltitol blend	45.6 g/day—2 weeks	*Bifidobacterium*	+	40	FISH	Beards et al. (73)
			*Bacteroides*	+			
			Lactobacilli	+			
			Eubacteria	+			
	
	Agave inulin	5.0/7.5 g/day—3 weeks	Actinobacteria	+	29	16S Illumina sequencing	Holscher et al. (83)
			*Bifidobacterium*	+			
			*Desulfovibrio*	−			
	
	Very long-chain inulin	10 g/day—3 weeks	*Bifidobacterium*	+	32	FISH	Costabile et al. (84)
			Lactobacilli-enterococci	+			
			*Atopobium*	+			
			*Bacteroides*–*Prevotella*	−			
	
	Inulin	10 g/day—16 days	*Bifidobacterium adolescentis*	+	12	qRT-PCR	Ramirez-Farias et al. (85)
			*Faecalibacterium prausnitzii*	+			
	
	Inulin/partially hydrolyzed guar gum	15 g/day—3 weeks	*Clostridium* sp.	−	60	RT-PCR + gas chromatography	Linetzky Waitzberg et al. (86)
	
	Soluble corn fiber	6 g/day—2 weeks	*Bifidobacterium*	+	24	FISH	Costabile et al. (24)
	
	Soluble corn fiber	12 g/day—2 weeks	*C. histolyticum*/ *C. perfringens*	−	24	FISH	Costabile et al. (24)
	
	Soluble corn fiber	21 g/day—3 weeks	*Firmicutes*	−	21	Whole-genome shotgun 454 pyrosequencing FLX-titanium	Holscher et al. (87)
			Bacteroidetes	+			
	
	Soluble corn fiber	10 g/day—4 weeks	*Parabacteroides*	+	27	16S Illumina sequencing	Whisner et al. (89)
	
	Soluble corn fiber	20 g/day—4 weeks	*Bacteroides*	−	27	16S Illumina sequencing	Whisner et al. (89)
			*Parabacteroides*	+			
			*Lachnospira*	−			
	
	Soluble corn fiber	21 g/day—3 weeks	*Bifidobacterium*	+	20	454 pyrosequencing FLX-titanium	Hooda et al. (88)
			Actinobacteria	−			
			Proteobacteria	+			

*The studies investigated the subsequent changes in gut microbiota composition*.

*^a^Total number of volunteers, divided over study groups*.

## *Bacteroides* 

Most studies demonstrated an increase in *Bacteroides* abundance by a variety of supplemental disaccharides, oligosaccharides, and polysaccharides, with doses ranging between 2.2 and 45.6 g per day, and duration between 2 and 10 weeks of treatment (73, 75, 76). Supplemental carbohydrates that reduced *Bacteriodes* were a very long-chain inulin given in a dose of 10 g/day over the course of 3 weeks (84) and soluble corn fiber given in a dose of 20 g/day over 4 weeks (89). Some studies, however, showed results that may be explained by other means than the supplemental carbohydrate per sé. For example, the study by Walton et al. (76) used a relatively low dose of arabinoxylan-oligosaccharides in bread with potential effects on *Bacteroides*, suggesting that its supplementation has a high feasibility for long-term human application. However, in that study, the increase in *Bacteroides* was also observed in the control group, presumably due to the higher total amount of (complex) carbohydrates present in the diet due to the control bread (76). Therefore, it is unknown whether arabinoxylan-oligosaccharides specifically are able to increase *Bacteroides* at this dose. The *Bacteroides*-increasing effect of 5.5 g per day by the mixture of galacto-oligosaccharides in the study by Vulevic et al. (75) was, therefore, more convincing.

If most prebiotics increase *Bacteroides* abundance (with the exception of very long-chain inulin), it is of interest to know whether this direction would be either beneficial or detrimental for subjects with obesity and cardiometabolic and/or inflammatory diseases. Studies investigating the association between *Bacteroides* and obesity and cardiometabolic health are abundant but highly contradicting (5, 92). Several studies have reported changes at the phylum level in relation to obesity, with a low abundance of Bacteroidetes relative to the proportion of Firmicutes in obese individuals (93, 94), as well as in genetically obese (ob/ob) mice (29, 95). For instance, a study by Armougom et al. (96) in 20 normal weight and 20 obese French individuals confirmed the decrease in Bacteroidetes abundance in obese individuals, accompanied by an increase of the genus *Lactobacillus*, belonging to the phylum Firmicutes. Studies by Turnbaugh et al. (97) and Furet et al. (98) only detected a reduced representation of Bacteroidetes, like *Bacteroides* and *Prevotella*, without changes in Firmicutes abundance in obese individuals. However, contradicting results were found in a study by Schwiertz et al. (99), where in overweight and obese subjects the ratio of Firmicutes to Bacteroidetes was increased in favor of the Bacteroidetes. Other findings even suggest that the proportions of Bacteroidetes and Firmicutes do not have a major relation with human obesity at all (100). At the genus level, a study investigating gut microbiota in pregnant women revealed a positive correlation between BMI before pregnancy and the abundance of *Bacteroides*. The high abundance of *Bacteroides* was also associated with excessive weight gain during pregnancy (101). Due to the higher number of studies reporting a decrease in Bacteroidetes in obesity, combined with the fact that some of these studies used 16S rRNA sequencing, which is more accurate than qPCR and FISH used in the contradicting studies, it is more likely that obesity is associated with a decrease in Bacteroidetes. However, at this point a causal role of Bacteroidetes or of the genus *Bacteroides* in obesity has not been convincingly proven.

One possible explanation for the above-mentioned indecisive link between *Bacteroides* and obesity is that it is not obesity *per se*, but the presence of adverse health conditions that are part of MetS that are closely associated with the abundance of *Bacteroides*. As mentioned before, MetS is often seen in obese individuals and is a risk factor for the development of cardiometabolic/inflammatory diseases (102). In a study by Haro et al. (22) on 239 volunteers of which 138 met the criteria for MetS, the relative abundance of *Bacteroides* indeed turned out to be higher in the MetS subjects than in subjects without MetS. The study did not report a relation between *Bacteroides* abundance and BMI or magnitude of obesity. Only a negative relation between waist circumference and the relative abundance of *Bacteroides thetaiotaomicron* was observed (*R* = 0.162, *P* = 0.022). It must be noted that the non-MetS group in this trial did not include healthy individuals, but individuals with a history of coronary heart disease that were involved in another clinical trial at the time (23). Furthermore, a higher abundance of *Bacteroides* has been reported to be associated with impaired glucose tolerance in T2DM patients as well (103). Finally, *Bacteroides* abundance was higher in individuals with several disease characteristics (but not necessarily marked as MetS) rendering them more at risk to develop pre-diabetes, T2DM, and CVD (104). While there are also studies showing no clear link between *Bacteroides* abundance and cardiometabolic diseases (105, 106), the available data on MetS and *Bacteriodes* abundance are exciting enough to emphasize on research investigating causal mechanisms between the two. It may for instance be speculated that obese individuals are more prone to develop MetS due to an already existing high abundance in *Bacteroides*, or alternatively, it could be that MetS in the host causes changes in *Bacteroides* abundance bidirectionally.

A pathological feature that is commonly observed in a wide range of chronic conditions such as MetS, non-alcoholic fatty liver disease, CVD, and T2DM is a persistent low-grade inflammatory state, characterized by increased levels of inflammatory cytokines (107–110). It is possible that the gut microbial imbalance and the permeability of the intestinal barrier contribute to this low-grade systemic inflammation, which may then be related to the onset and progression of cardiometabolic derangements (111). However, it could also be the other way around, being that the state of inflammation is the cause of the microbial dysbiosis. Interestingly, also higher abundances of *Bacteroides* species were found in patients with IBD compared to healthy individuals (112, 113), suggesting that a disturbance of the gut microbial balance in favor of *Bacteroides* is linked to inflammation of the GI tract. IBD is relatively frequently associated with MetS (114, 115), which adds to the possibility that *Bacteroides* abundance and disturbances in the inflammatory system are linked. The direction of this relation is currently unclear.

## *Clostridium* 

*Clostridium* levels were reported to decrease by the supplementation of 65.2 g of raffinose/stachyose for 2 weeks (74), by 15 g of a mixture of inulin and partially hydrolyzed guar gum during a 3-week supplemental intervention study (86), or by supplementation with 5 g of α-galacto-oligosaccharides for 2 weeks (91). Of the previous studies, the latter has a highest feasibility due to the lower dose. Furthermore, it must be noted that the study supplementing with a mixture of inulin and partially hydrolized guar gum investigated the matter in constipated women, leading to an increased transit time wherefore the effect could be different in individuals without constipation.

The decrease of *Clostridium* abundance has been linked to decreased severity of T2DM and obesity (101, 105, 116). For example, in the studies by Karlsson et al. (105) and Larsen et al. (116), lower levels of the genus *Clostridium* and the class Clostridia, respectively, were found in T2DM patients compared to healthy controls. In addition, an inverse relation between *Clostridium* genus abundance and high-sensitivity C-reactive protein (hs-CRP), an inflammatory marker associated with the risk of CVD (26), has been observed (117). This suggests a potentially beneficial link between abundance of the *Clostridium* genus and metabolic health. Moreover, the expansion of Clostridia species belonging to *Clostridium* cluster IV and XIVa after the conventionalization of germ-free mice coincided with increasing concentrations of SCFAs, such as acetate, propionate, and butyrate (118), of which the beneficial effects have been discussed before. These latter studies all describe beneficial effects of the presence of specific *Clostridium* species. However, studies reporting higher T2DM and CVD risk with lowered *Clostridium* levels are available as well (119, 120). Due to the controversy, it remains unclear whether the decrease in *Clostridium* that could possibly be established by the consumption of supplemental dietary carbohydrates is beneficial for health.

One explanation for this contradiction could be the accuracy of the detection methods employed to assess the composition of microbiota. The majority of these studies reveal difference on phylum or genus level, which fails to differentiate between commensal and opportunistic Clostridia. Indeed, studies assessing health effects of *Clostridium* that focus on the species level reported high levels of opportunistic pathogens like *Clostridium hathewayi* and *Clostridium ramosum* to be linked to T2DM (106), whereas high weight loss was associated with a decrease in *Clostridium coccoides* and *histolyticum* (121, 122). In addition, *Clostridium difficile* is well known for its infectious properties, as it is an important cause of diarrhea in hospitalized patients (123). Finally, the presence of *Clostridium septicum* is suspected to play a role in colorectal carcinogenesis (124).

Altogether, there is no clear answer to the question how changes in *Clostridium* affect health, mainly because at the species level changes in abundance are known to have a great variety of effects. Moreover, the *Clostridium* genus includes species with a rather widespread evolutionary distribution, based on which this genus is not considered a phylogenetically coherent taxon (125). This high diversity in species within this genus makes it hard and possibly unreliable to identify health effects of the genus as a whole.

## *Lactobacillus* 

Merely increases in Lactobacilli abundance were reported by supplementation with disaccharides, oligosaccharides, and polysaccharides (73, 74, 76, 84, 91). In cases where changes in Bacteroidetes and *Lactobacillus* counts were found in the same study, their abundance increased, except for the study by Costabile et al. (84), where *Lactobacillus enterococci* abundance increased, whereas the *Bacteroides* and *Prevotella* counts decreased by supplementation of very long-chain inulin supplementation. In general, an increase in *Lactobacillus* abundance by supplemental dietary carbohydrates may be of potential interest since levels have been inversely associated with obesity (119, 122, 126). For example, the prevalence of the species *Lactobacillus plantarum* was higher in a group of lean versus obese individuals (76 versus 27%, *p* = 0.005) (119). Weight loss in overweight adolescents due to a calorie-restricted diet and increased physical activity has been reported to be associated with an increase in Lactobacilli abundance (122). In addition, a study in obese rats showed that *Lactobacillus* abundance was increased upon controlled exercise training (i.e., which is often associated with weight loss) (126). Contradicting results have been mentioned too. Armougom et al. (96) observed significantly higher *Lactobacillus* species concentrations in adult obese subjects compared to lean ones. The same was recently reported for obese children (127). With qPCR as technique used in these studies, as well as in two out of three studies reporting an inverse relation between *Lactobacillus* and obesity, it is unlikely that the contradicting results are caused by differences in microbiota assessment.

A study by Karlsson et al. (105) in European women with normal, impaired, and diabetic glucose control revealed a positive association between *Lactobacillus* abundance and blood glucose levels by making use of metagenome-wide Illumina sequencing. Similar results were found in two qPCR studies in Japanese and Danish adults with T2DM, when compared to healthy individuals (116, 120). In addition, higher *Lactobacillus* abundance was found in participants diagnosed with MetS (23). These findings support the study by Armougom et al. (96) suggesting a positive association of high abundance of Lactobacilli with obesity and its implications.

There is nevertheless a plethora of studies showing that probiotic treatment (with or without supplemental dietary carbohydrates) with Lactobacilli strains could potentially be useful to combat obesity and cardiometabolic derangements. For instance, yogurt containing Lactobacilli led to decreased fasting plasma glucose and improved antioxidant status (and thus may have the potential to treat and/or prevent T2DM), which matches the previous studies in support of the benefits of increased *Lactobacillus* (128). Second, a study by Sanchez et al. in which capsules containing *Lactobacillus rhamnosus* and a mixture of inulin and oligofructose were ingested twice a day for a period of 24 weeks by obese women was able to cause better sustained weight maintenance after a period of weight loss than controls (129). Because of the fact that supplemental dietary carbohydrates often lead to an increase in *Lactobacillus* as well as in *Bacteroides* abundance, the latter effect may potentially abolish the positive effect of *Lactobacillus*.

## *Bifidobacterium* 

Abundance of *Bifidobacterium* increased upon supplementation with disaccharides, oligosaccharides, and polysaccharides (64, 73–75, 77–85, 88, 91). Supplementation with xylo-oligosaccharide seemed to have the highest feasibility, since only 1.4 g per day was found to be sufficient to increase *Bifidobacterium* abundance, with a treatment duration of 8 weeks (64). Xylo-oligosaccharide is naturally present in vegetables, fruits, milk, and honey, and can also be produced industrially (64).

The effects of the *Bifidobacterium* abundance on the host’s health have been well established. A study by Teixeira et al. (119) investigated whether the abundance of specific microbes was associated with anthropometric, body composition, and biochemical measurements. The study was performed in 20 lean and 20 obese Brazilian women in their late twenties or early thirties. *Bifidobacterium* genus counts were found to be significantly higher in lean women. In addition, Bifidobacteria abundance appeared to be inversely associated with insulin homeostasis model assessment (HOMA) index. The low abundance of Bifidobacteria in overweight adults and children, as well as in women gaining relatively much weight during pregnancy, has been reported in other studies as well (20, 101, 122, 130, 131). The inverse relation between Bifidobacteria abundance and diabetes was also found in a cohort study of 50 Chinese T2D patients compared to 30 healthy Chinese individuals (132). Comparable findings were reported in mice (133).

Plasma levels of bacterial lipopolysaccharide (LPS), a cell wall constituent of gram-negative bacteria with potent inflammatory actions (108), are inversely related to *Bifidobacterium* abundance in the colon. Because chronic treatment with LPS in rodents is able to induce obesity and insulin resistance (134), there may be a course of events that link LPS, *Bifidobacterium* abundance, obesity, and inflammation (134, 135). The underlying mechanism may lie in the fact that Bifidobacteria is known to be involved in the maintenance of the mucosal barrier (136, 137). Two possible scenarios may explain the link between *Bifidobacterium* loss and obesity and inflammation could be explained. Loss of *Bifidobacterium* may lead to higher transit of LPS and other bacterial fragments across the gut into systemic circulation, resulting in a state of high plasma LPS. This state is referred to as metabolic endotoxemia and is frequently observed in obesity (20, 134). The second scenario is that this mechanism might be preceded by inflammation, which would enable certain members of the microbiota to survive the environment, while others would not. These surviving members will mainly be pathobionts, resulting in a microbial dysbiosis leading to a washout of beneficial members like Bifidobacteria (138). A breach in the epithelial layer as a result of inflammation could subsequently lead to the transfer of LPS across the epithelial layer into the blood circulation, resulting in endotoxemia. Also other pathways exist that may not require LPS, which have been reviewed by others (139, 140).

Aside from the relation between *Bifidobacterium* counts and the prevalence of obesity, MetS, and T2DM, the potential of Bifidobacteria in the treatment of T2DM came to light more recently. A study by Tonucci et al. (141) in 50 Brazilian volunteers with T2DM (BMI <35 kg/m^2^, aged 35–60 years) revealed that treatment with fermented milk containing *Bifidobacterium lactis* and *Lactobcillus acidophilus* was able to improve glycemic control and decrease inflammatory markers. Similar findings have been reported by other studies in humans as well (128, 142). In contrast to these findings, however, two studies in Estonia revealed a relation between higher *Bifidobacterium* abundance and higher blood glucose levels and occurrence of obesity in elderly and preschool children, respectively (143, 144). Bacteriological evaluation of the fecal samples in these studies, however, was based on manual counting of bacterial colony forming units on different agar plates, a method that is prone to errors (71, 145). Therefore, these contradicting conclusions are not highly reliable.

Overall, the effects of complex dietary supplemental carbohydrates to increase abundance of *Bifidobacterium* are useful since there is consensus that abundance of *Bifidobacterium* is lower in subjects who are obese and have cardiometabolic and/or inflammatory diseases.

## *Atopobium* 

The *Atopobium* genus was reported to be increased by disaccharides and polysaccharides, with a treatment of 10–45.6 g a day for 2–3 weeks (73, 84). Very long-chain inulin was able to establish the increase within three weeks at a dose of 10 g a day, which seems a feasible dose (84). *Atopobium* is a genus belonging to the class Actinobacteria, the class to which Bifidobacteria belongs to as well. How *Atopobium* is related to health is only reported in a limited number of studies, but all report favorable effects of higher *Atopobium* abundance. The genus is relatively unknown and only four studies were found investigating the relation of *Atopobium* to health. The first study by Sato et al. (120) reported lower counts of the *Atopobium* cluster in 50 Japanese T2DM patients, compared to 50 healthy individuals. This increase in *Atopobium* abundance could potentially play a role in the prevalence of T2DM. In addition, the study found a negative correlation of *Atopobium* abundance with levels of the inflammation marker hs-CRP and BMI, and a positive correlation with HDL cholesterol. These correlation all support the beneficial effects of increased *Atopobium* counts on cardiometabolic health. A second study by Maccaferri et al. (146) investigated changes in the gut microbiota composition upon treatment with Rifaximin, a drug which is suggested to be able to induce clinical remission of active Crohn’s disease, which belongs to the cluster of IBDs. The study in an *in vitro* human colonic model system revealed that *Atopobium* levels were increased upon treatment, suggesting that increasing *Atopobium* could be part of the underlying mechanisms of the Rifaximin-induced reduction of inflammation of the bowel. Finally, Khachatryan et al. (147) analyzed the composition of the gut microbiota in patients with familial Mediterranean fever (FMF), an auto-inflammatory disease characterized by recurrent self-resolving attacks of fever and polyserositis, followed by periods of remission without clinical signs. In that study, a trend was observed in which *Atopobium* was less abundant in the gut microbiota of FMF patients compared to healthy controls, where abundance was lowest in attack and slightly higher during remission (42.2 counts per gram of fecal specimen for healthy individuals, 28.9 in remission, and 16.9 in attack, where this last value was significantly lower compared to the counts in healthy controls).

In conclusion, not many studies investigating the link between *Atopobium* abundance and cardiometabolic health are available, but the available ones discussed here do point in the direction of a beneficial effect of *Atopobium* abundance.

## *Desulfovibrio* 

*Desulfovibrio* abundance was reported to be decreased by 5.0 g of agave inulin intake per day for 21 days (83). Similar findings were reported in obese subjects supplemented with a mixture of galacto-oligosaccharides (148). In a Chinese cohort, this genus, that includes sulfate-reducing bacteria, has been reported to be increased in T2DM patients (106). In addition, a study in mice that were fed a diet supplemented with glycomacropeptide, a 64-amino acid glycophosphopeptide, showed that this diet reduced *Desulfovibrio* bacteria. This reduction was associated with decreased plasma concentrations of the inflammation markers interferon-gamma, tumor necrosis factor-alpha, Interleukin-1beta, and Interleukin-2. These changes could contribute to the management of obesity, IBD, and phenylketonuria (i.e., a disease due to impaired metabolism of the amino acid phenylalanine) (149). This finding perhaps shares some grounds with the increased presence of *Desulfovibrio* species in patients suffering from ulcerative colitis, a type of IBD (150).

The aforementioned studies are all in favor of the hypothesis that lowered *Desulfovibrio* abundance can benefit health. However, the one study by Karlsson et al. (151) contradicts this because they reported lowered levels of *Desulfovibrio* in overweight and obese children in the age of 4–5 years, compared to children with a normal BMI. This contradiction might be age-related as the other studies investigating the *Desulfovibrio* abundance in humans all involved older participants.

## The Choice of Supplemental Fermentable Carbohydrates

Based on the data discussed above, it appears that dietary carbohydrate supplementation in healthy individuals seems to be associated with increases in the abundance of *Bacteroides, Lactobacillus, Bifidobacterium*, and *Atopobium*, whereas lower levels of abundance were generally observed in *Clostridium* and *Desulfovibrio*. With respect to the health effects of *Bacteroides*, there are discrepancies between obesity on the one hand and the cardiometabolic/inflammatory derangements that are often associated with it on the other hand. Thus, while both lower levels and higher levels of *Bacteroides* are reported to be found in obese individuals, higher levels seem to be associated primarily with the occurrence of MetS, T2DM, and IBD, all derangements known to involve increased activity of the inflammatory system (107–109). For this reason, it seems counterproductive to treat obese individuals, particularly those with MetS, and/or related inflammatory diseases with dietary supplemental carbohydrates that would augment *Bacteroides* abundance. Dietary supplemental carbohydrates that do not increase the abundance of *Bacteroides*, but Bifidobacteria and potentially also *Lactobacillus* (although it is not yet clear about the latter whether it contributes to or protects against the onset of obesity, but see discussion on this subject below) instead, appear more helpful. Inulin, for example, may fulfill the above-mentioned criteria, as it increases Bifidobacteria abundance, and in some cases reduces *Bacteroides* abundance. Several papers show positive effects of inulin supplementation to reduce the severity of cardiometabolic and inflammatory derangements in obese and diabetic patients (42, 152). Also several animal studies point in this direction. For example, Rault-Nania et al. (153) showed that long-chain inulin supplementation in the diet of Wistar rats was able to reduce hypertriglyceridemia, hypertension, and heart peroxidation (i.e., increases in these are all signs of impaired cardiometabolic health) as a result of feeding a high fructose diet. Furthermore, mice exposed to a high-fat diet for 8 weeks with supplemental inulin (10%) or β-glucan (10%) had lower body weight gain relative to controls, with a lower energy intake observed in β-glucan supplemented mice than in inulin supplemented mice. In addition, the latter group showed reduced body fat content, but the former presented stronger hypothalamic neuronal effects. Fecal/cecal *Bifidobacterium* and *Lactobacillus* abundance was increased in both supplemental groups (154). The effect of inulin supplementation on cardiometabolic health was also assessed in gnotobiotic mice colonized with simplified human microbiota and challenged with a high-fat diet. Although the degree of obesity induction in these mice was comparable to control mice subjected to a high-fat diet, the inulin supplemented mice had increased bacterial diversity (including those of *Bifidobacterium* counts), significantly elevated concentrations of total SCFA in cecum and portal vein plasma, signs of reduced lipogenesis, and an increased ratio of plasma and liver omega-3 versus omega-6 fatty acids (155). A very recent paper by Salazar et al. (156) indeed showed that inulin-type fructan supplementation of 16g/day over a 3-month period in 30 obese women (i.e., with maltodextrine as control treatment) caused significant fecal increases in several *Bifidobacterium* species, with *Bifidobacterium longum* being negatively correlated with serum LPS endotoxin levels. In addition, fasting insulinemia and HOMA index were significantly lower in the supplemented group than in the placebo group after the treatment period, albeit that SCFA, acetate and propionate concentrations also declined.

An important point that deserves more attention is the dose of the dietary carbohydrate supplementation needed to establish the alterations in gut microbiota, since some of the reported studies used doses of which long-term tolerability is questionable. For example, a dose–response study by Bruhwyler et al. (157) showed that a dose of 20 g of inulin per day resulted in gastrointestinal complaints like flatulence. However, the cutoff of tolerability will likely differ per prebiotic and individual. Also the method of application may be of importance as it has been reported that soluble corn fiber is well tolerated up to 65 g/day, but only when consumed in multiple doses (158). Related to this point is the fact that none of the studies reporting on gut microbiota changes by supplemental carbohydrates assessed the regular dietary micro-/macronutrient and fiber composition of the participants. Since it is clear that the composition of the diet can affect the composition of the gut microbiota, this is a possible factor underlying the reported variability in responses and sometimes contradicting results between studies.

## Conclusion and Gaps in Understanding

Whereas it is relatively clear in which direction supplemental fermentable carbohydrates can alter the gut microbiome, the relevance of these changes regarding health remains controversial. As already mentioned above, a clear exception is the genus *Bifidobacterium*, of which the benefits of an increased abundance have been well established. Increases in *Lactobacillus* by dietary supplemental carbohydrates could potentially be health promoting as well (i.e., given the plethora of studies showing that probiotics that increase Lactobacilli generally can counter obesity and cardiometabolic diseases) but these effects may be counteracted by the stimulatory effects on *Bacteroides* abundance that often associates the effects on *Lactobacillus*. The controversy regarding *Bacteroides* and its role in obesity is also related to the discussion regarding the ratio between the phyla Bacteroidetes and Firmicutes, which has been reported to be related to obesity numerous times. However, the studies do not agree upon which of the phyla is the higher abundant phylum in obese individuals. The answer is still indefinite, indicating that it concerns a very complex matter of which not all the details are known yet. Various health effects are reported at the species level, but the diversity of species within a genus makes that it is difficult to draw conclusions at the genus level. Therefore, there is a need for more comprehensive and species-specific characterization of the human microbiome. Studying the meta-transcriptome may be the most informative when linked also to either a species level picture of the microbiome or the genetic profile of the population as a whole. The difficulty here of course is that we know the function of so few of the genes (i.e., not only what they mean for functioning of the specific bacteria but also for the host) but this knowledge is expected to grow quickly.

The majority of studies performed on obesity and microbiota ignore the fact that different causes of obesity should be taken into account. In short, maintenance of body weight is the result of a sustained match between energy intake and energy expenditure, and increased intake of energy-dense foods and/or a decreased physical activity can logically underlie weight gain (94). Resistance to the adipocyte hormone leptin has been mentioned to be the resultant of the above-mentioned mismatch (159), or even the cause (140), which both could also explain the associated co-morbidities (160). Differences in energy-harvesting efficiency of microbiota (as a result of variation in its composition and abundance) can also play a role in the energy balance or imbalance herein, and the latter may contribute significantly to weight gain as well (2, 9, 94, 161, 162). As with recording of the habitual diet, also none of the studies reported in this review have assessed the energy budgets of the participants, which thus obstructs further insight into the contribution of energy absorption versus expenditure in the resultant body fat. Designing a study in which a large cohort of obese individuals with and without MetS (or related non-communicable diseases) is further divided into subgroups based on their energy budget, with one group having a more positive energy budget than the other, and subsequently investigate whether gut microbiota abundance and diversity between these subgroups differs at baseline would be of high relevance. Subsequent supplementation with dietary carbohydrates aiming to relief obesity and/or cardiometabolic/inflammatory diseases should then include investigation of the entire energy balance equation in these individuals.

Weight gain in the form of increasing adiposity stores (i.e., which is a key risk factor to attract cardiometabolic and inflammatory health problems) is probably the result of selective or evolutionary pressures that have shaped our paleolithic (or even earlier) ancestors to deal with food shortages. This theory was first hypothesized by Neel to explain the current epidemic of diabetes (163), and was later expanded toward the epidemic of obesity [see Ref. (164) as an example]. The above-mentioned evolutionary pressure probably also co-shaped the gut microbiome (165, 166). To this end, it is of interest to consider the possibility that the gut microbiota affects the hosts’ (and therefor its own) energy balance to increase the chances of survival in times of famine by offering ample nutrients from otherwise indigestible food ingredients, but also—via the microbiota-gut-brain axis—to affect the host’s eating behavior, hunger/satiety, and even reward and mood aspects of food, to stimulate food searching, craving, etc. (167). In our current western-industrialized society, where industrialized and processed foods are highly abundant, but contain relatively little amounts of fibers, this may underlie a conflict between host and microbiota leading to increased craving as well (167). Recent findings in line with this hypothesis are reported by Perry et al. (168). This study in rats revealed that exposure to a calorically dense high-fat diet resulted in increased ghrelin secretion and glucose-stimulated insulin secretion, which may promote hyperphagia and increased energy storage as fat. The reported changes were found to be the result of an altered gut microbiota, acting through the parasympathetic nervous system. For this reason, a primal or ancestral diet, which contains in itself many of the complex carbohydrates with prebiotic properties as discussed in this review, should be promoted more rigorously in order to prevent obesity and cardiometabolic/inflammatory diseases [e.g., by restoring leptin sensitivity (140)]. These sorts of mechanisms are of interest from an “evolutionary medicine” point of view, and should certainly be taken into account to fully understand the role of the microbiome in health and disease.

## Future Directions

Although there is consensus that supplemental dietary carbohydrates have the tendency to increase the abundance of *Bifidobacterium, Bacteroides*, Lactobacilli, and *Atopobium* and to decrease the abundance of *Clostridium* and *Desulfovibrio* in the human gut, it remains unclear whether specific types of fermentable carbohydrates target specific bacterial groups, or whether a change in abundance of one group would influence the rest of the community through cross feeding. That being said, the definition of prebiotics as it is currently used—“non-digestible food ingredients that beneficially affect the host by selectively stimulating the growth and/or activity of one or a limited number of bacterial species already resident in the colon, in the attempt to improve host health” (36)—might need revision. The stimulation of beneficial bacterial species might in fact be mediated by or coincided with a decrease or increase in abundance of a species with undetermined health effects. Therefore, a suggested alternative definition of prebiotics would be “non-digestible food ingredients that affect the host by selectively stimulating or inhibiting the growth and/or activity of one or a limited number of bacterial species already resident in the colon, with the net effect of the microbial change being beneficial for the host’s health.” It might not be reliable to generalize the effects of prebiotics since this will likely depend on the host genotype, current health status, and susceptibility to disease (169). How then alterations in gut microbiota exactly influence the host’s health remains largely undetermined, but several mechanisms may contribute as already explained in the previous parts of this review and elsewhere (140, 170).

The gut microbiota carry out a wide range of biotransformation reactions during metabolism of the diet, including those that are not present in the mammalian host (171). This provides an important way to further investigate the effect of changes in the gut microbiota, established by prebiotics, on the host’s health, i.e., by means of investigating the microbiota-dependent metabolites. Trimethylamine, for example, is the product of microbiota-dependent choline (essential dietary nutrient) metabolism (172). A potential link between the intestinal microbiota, dietary choline, and CVD risk has been suggested, where increased metabolites of the dietary lipid phosphatidylcholine have been observed in the serum of patients with CVD pathogenesis (19, 173, 174). This makes that these metabolites are potential measures to assess health effects of changes in microbial genera discussed in this review, but applicability and reliability of this measure must be elucidated first. This research area of mass spectrometry identified signatures is quickly expanding and candidate metabolite factors are increasingly identified with notable bioactivities (175). With the current knowledge, however, it remains hard to determine whether alterations in gut microbiota and/or their metabolites are cause or consequence of cardiometabolic/inflammatory diseases. Thus, while it has been confirmed that obesity, MetS, T2DM, and CVD are usually associated with chronic inflammation (176) and convincing evidence links the gut microbiome to inflammation (177–179), future research should focus on whether, how and to what extent mechanisms of inflammation and beyond are indeed bidirectional between the host and the microbiome. This requires system biological approaches and “big-data” analysis combined with in depth analysis of the effect of targeted perturbations in microbiota as well as in the host. Refined studies on the effects of reconstitution of microbiota in gnotobiotic mouse systems may be important for unraveling some of these interactions. Human intestinal organoids (i.e., near physiological 3D models of the human gut grown from human stem cells) appear to have great potential for studying these interactions as well (180) and eventually may take this to a “personalized” therapeutic level (181), from the perspective of both the host and the microbiome.

## Author Contributions

All authors were involved in designing and planning this paper. PV wrote the first drafts that were corrected and added by SA and GD. GD edited the final versions. All authors take public responsibility for the content of the article regarding data interpretation. They do not have any conflict of interest.

## Conflict of Interest Statement

The authors declare that the research was conducted in the absence of any commercial or financial relationships that could be construed as a potential conflict of interest.

## References

[1] Purchiaroni F Tortora A Gabrielli M Bertucci F Gigante G Ianiro G The role of intestinal microbiota and the immune system. Eur Rev Med Pharmacol Sci (2013) 17:323–33. 23426535

[2] El Aidy S van Baarlen P Derrien M Lindenbergh-Kortleve DJ Hooiveld G Levenez F Temporal and spatial interplay of microbiota and intestinal mucosa drive establishment of immune homeostasis in conventionalized mice. Mucosal Immunol (2012) 5:567–79. 10.1038/mi.2012.32 22617837

[3] Bird AR Brown IL Topping DL. Starches, resistant starches, the gut microflora and human health. Curr Issues Intest Microbiol (2000) 1:25–37. 11709851

[4] El Kaoutari A Armougom F Gordon JI Raoult D Henrissat B. The abundance and variety of carbohydrate-active enzymes in the human gut microbiota. Nat Rev Microbiol (2013) 11:497–504. 10.1038/nrmicro3050 23748339

[5] Tagliabue A Elli M. The role of gut microbiota in human obesity: recent findings and future perspectives. Nutr Metab Cardiovasc Dis (2013) 23:160–8. 10.1016/j.numecd.2012.09.002 23149072

[6] Eckburg PB Bik EM Bernstein CN Purdom E Dethlefsen L Sargent M Diversity of the human intestinal microbial flora. Science (2005) 308:1635–8. 10.1126/science.1110591 15831718 PMC1395357

[7] Goodrich JK Waters JL Poole AC Sutter JL Koren O Blekhman R Human genetics shape the gut microbiome. Cell (2014) 159(4):789–99. 10.1016/j.cell.2014.09.053 25417156 PMC4255478

[8] Penders J Thijs C Vink C Stelma FF Snijders B Kummeling I Factors influencing the composition of the intestinal microbiota in early infancy. Pediatrics (2006) 118:511–21. 10.1542/peds.2005-2824 16882802

[9] Johnson CL Versalovic J. The human microbiome and its potential importance to pediatrics. Pediatrics (2012) 129:950–60. 10.1542/peds.2011-2736 22473366 PMC3340594

[10] Gur TL Bailey MT Effect of stress on commensal microbes and immune system activity. Adv Exp Med Biol (2016) 874:289–300. 10.1007/978-3-319-20215-0_14 26589225

[11] O’Mahony SM Marchesi JR Scully P Codling C Ceolho AM Quigley EM Early life stress alters behavior, immunity, and microbiota in rats: implications for irritable bowel syndrome and psychiatric illnesses. Biol Psychiatry (2009) 65:263–7. 10.1016/j.biopsych.2008.06.026 18723164

[12] Mikkelsen KH Allin KH Knop FK Effect of antibiotics on gut microbiota, glucose metabolism and bodyweight regulation – a review of the literature. Diabetes Obes Metab (2016) 18:444–53. 10.1111/dom.12637 26818734

[13] Agans R Rigsbee L Kenche H Michail S Khamis HJ Paliy O. Distal gut microbiota of adolescent children is different from that of adults. FEMS Microbiol Ecol (2011) 77:404–12. 10.1111/j.1574-6941.2011.01120.x 21539582 PMC4502954

[14] Claesson MJ Jeffery IB Conde S Power SE O’Connor EM Cusack S Gut microbiota composition correlates with diet and health in the elderly. Nature (2012) 488:178–84. 10.1038/nature11319 22797518

[15] Mariat D Firmesse O Levenez F Guimarăes V Sokol H Doré J The firmicutes/bacteroidetes ratio of the human microbiota changes with age. BMC Microbiol (2009) 9:123. 10.1186/1471-2180-9-123 19508720 PMC2702274

[16] Vanhoutte T De Preter V De Brandt E Verbeke K Swings J Huys G. Molecular monitoring of the fecal microbiota of healthy human subjects during administration of lactulose and *Saccharomyces boulardii*. Appl Environ Microbiol (2006) 72:5990–7. 10.1128/AEM.00233-06 16957220 PMC1563651

[17] Zoetendal EG Akkermans AD De Vos WM. Temperature gradient gel electrophoresis analysis of 16S rRNA from human fecal samples reveals stable and host-specific communities of active bacteria. Appl Environ Microbiol (1998) 64:3854–9. 9758810 10.1128/aem.64.10.3854-3859.1998 PMC106569

[18] David LA Maurice CF Carmody RN Gootenberg DB Button JE Wolfe BE Diet rapidly and reproducibly alters the human gut microbiome. Nature (2014) 505(7484):559–63. 10.1038/nature12820 24336217 PMC3957428

[19] Aron-Wisnewsky J Clément K. The gut microbiome, diet, and links to cardiometabolic and chronic disorders. Nat Rev Nephrol (2016) 12(3):169–81. 10.1038/nrneph.2015.191 26616538

[20] Shen J Obin MS Zhao L. The gut microbiota, obesity and insulin resistance. Mol Aspects Med (2013) 34:39–58. 10.1016/j.mam.2012.11.001 23159341

[21] Zhang C Zhang M Wang S Han R Cao Y Hua W Interactions between gut microbiota, host genetics and diet relevant to development of metabolic syndromes in mice. ISME J (2010) 4:232–41. 10.1038/ismej.2009.112 19865183

[22] Haro C Montes-Borrego M Rangel-Zúñiga OA Alcalá-Díaz JF Gómez-Delgado F Pérez-Martínez P Two healthy diets modulate gut microbial community improving insulin sensitivity in a human obese population. J Clin Endocrinol Metab (2016) 101(1):233–42. 10.1210/jc.2015-3351 26505825

[23] Haro C Garcia-Carpintero S Alcala-Diaz JF Gomez-Delgado F Delgado-Lista J Perez-Martinez P The gut microbial community in metabolic syndrome patients is modified by diet. J Nutr Biochem (2015) 27:27–31. 10.1016/j.jnutbio.2015.08.011 26376027

[24] Costabile A Deaville ER Morales AM Gibson GR Prebiotic potential of a maize-based soluble fibre and impact of dose on the human gut microbiota. PLoS One (2016) 11(1):e0144457 10.1371/journal.pone.0144457 26731113 PMC4701468

[25] Cummings JH Stephen AM Carbohydrate terminology and classification. Eur J Clin Nutr (2007) 61(Suppl 1):S5–18. 10.1038/sj.ejcn.1602936 17992187

[26] Hansen TH Gøbel RJ Hansen T Pedersen O. The gut microbiome in cardio-metabolic health. Genome Med (2015) 7:33. 10.1186/s13073-015-0157-z 25825594 PMC4378584

[27] Matheus AS Tannus LR Cobas RA Palma CC Negrato CA Gomes MB. Impact of diabetes on cardiovascular disease: an update. Int J Hypertens (2013) 2013:653789. 10.1155/2013/653789 23533715 PMC3603160

[28] McCarthy MI Genomics, type 2 diabetes, and obesity. N Engl J Med (2010) 363:2339–50. 10.1056/NEJMra0906948 21142536

[29] Turnbaugh PJ Ley RE Mahowald MA Magrini V Mardis ER Gordon JI. An obesity-associated gut microbiome with increased capacity for energy harvest. Nature (2006) 444:1027–31. 10.1038/nature05414 17183312

[30] Bruce-Keller AJ Salbaum JM Luo M Blanchard E IV Taylor CM Welsh DA Obese-type gut microbiota induce neurobehavioral changes in the absence of obesity. Biol Psychiatry (2015) 77:607–15. 10.1016/j.biopsych.2014.07.012 25173628 PMC4297748

[31] Ridaura VK Faith JJ Rey FE Cheng J Duncan AE Kau AL Cultured gut microbiota from twins discordant for obesity modulate adiposity and metabolic phenotypes in mice. Science (2013) 341:6150 10.1126/science.1241214 PMC3829625 24009397

[32] Turnbaugh PJ Bäckhed F Fulton L Gordon JI. Diet-induced obesity is linked to marked but reversible alterations in the mouse distal gut microbiome. Cell Host Microbe (2008) 3:213–23. 10.1016/j.chom.2008.02.015 18407065 PMC3687783

[33] Bäckhed F Ding H Wang T Hooper LV Koh GY Nagy A The gut microbiota as an environmental factor that regulates fat storage. Proc Natl Acad Sci U S A (2004) 101:15718–23. 10.1073/pnas.0407076101 15505215 PMC524219

[34] Bäckhed F Manchester JK Semenkovich CF Gordon JI. Mechanisms underlying the resistance to diet-induced obesity in germ-free mice. Proc Natl Acad Sci U S A (2007) 104:979–84. 10.1073/pnas.0605374104 17210919 PMC1764762

[35] Bull MJ Plummer NT Part 1: the human gut microbiome in health and disease. Integr Med (Encinitas) (2014) 13:17–22. 26770121 PMC4566439

[36] Gibson GR Roberfroid MB. Dietary modulation of the human colonic microbiota: introducing the concept of prebiotics. J Nutr (1995) 125:1401–12. 7782892 10.1093/jn/125.6.1401

[37] Kellow NJ Coughlan MT Reid CM. Metabolic benefits of dietary prebiotics in human subjects: a systematic review of randomised controlled trials. Br J Nutr (2014) 111:1147–61. 10.1017/S0007114513003607 24230488

[38] Ghouri YA Richards DM Rahimi EF Krill JT Jelinek KA DuPont AW. Systematic review of randomized controlled trials of probiotics, prebiotics, and synbiotics in inflammatory bowel disease. Clin Exp Gastroenterol (2014) 7:473–87. 10.2147/CEG.S27530 25525379 PMC4266241

[39] Dewulf EM Cani PD Claus SP Fuentes S Puylaert PG Neyrinck AM Insight into the prebiotic concept: lessons from an exploratory, double blind intervention study with inulin-type fructans in obese women. Gut (2013) 62:1112–21. 10.1136/gutjnl-2012-303304 23135760 PMC3711491

[40] de Luis DA de la Fuente B Izaola O Conde R Gutiérrez S Morillo M Double blind randomized clinical trial controlled by placebo with an alpha linoleic acid and prebiotic enriched cookie on risk cardiovascular factor in obese patients. Nutr Hosp (2011) 26:827–33. 10.1590/S0212-16112011000400024 22470031

[41] Kellow NJ Coughlan MT Savige GS Reid CM. Effect of dietary prebiotic supplementation on advanced glycation, insulin resistance and inflammatory biomarkers in adults with pre-diabetes: a study protocol for a double-blind placebo-controlled randomised crossover clinical trial. BMC Endocr Disord (2014) 14:55. 10.1186/1472-6823-14-55 25011647 PMC4099169

[42] Dehfhan P Gargari BP Jafar-Abadi MA Aliasgharzadeh A. Inulin controls inflammation and metabolic endotoxemia in women with type 2 diabetes mellitus: a randomized-controlled clinical trial. Nutrition (2014) 30:418–23. 10.1016/j.nut.2013.09.005 24059649

[43] Giacco R Clemente G Luongo D Lasorella G Fiume I Brouns F Effects of short-chain fructo-oligosaccharides on glucose and lipid metabolism in mild hypercholesterolaemic individuals. Clin Nutr (2004) 23:331–40. 10.1016/j.clnu.2003.07.010 15158296

[44] Fransen F Sahasrabudhe N Elderman M Bosveld M El Aidy S Hugenholtz F ß2>1-fructans modulate the immune system in vivo by direct interaction with the mucosa in a microbiota-independent fashion. Front Immunol (2017) 8:154 10.3389/fimmu.2017.00154 28261212 PMC5311052

[45] den Besten G van Eunen K Groen AK Venema K Reijngoud DJ Bakker BM. The role of short-chain fatty acids in the interplay between diet, gut microbiota, and host energy metabolism. J Lipid Res (2013) 54:2325–40. 10.1194/jlr.R036012 23821742 PMC3735932

[46] Martinez RCR Bedani R Saad SMI Scientific evidence for health effects attributed to the consumption of probiotics and prebiotics: an update for current perspectives and future challenges. Br J Nutr (2015) 114:1993–2015. 10.1017/S0007114515003864 26443321

[47] Grider JR Piland BE. The peristaltic reflex induced by short-chain fatty acids is mediated by sequential release of 5-HT and neuronal CGRP but not BDNF. Am J Physiol Gastrointest Liver Physiol (2007) 292:G429–37. 10.1152/ajpgi.00376.2006 16973914

[48] Chakraborti CK. New-found link between microbiota and obesity. World J Gastrointest Pathophysiol (2015) 6:110. 10.4291/wjgp.v6.i4.110 26600968 PMC4644874

[49] Yadav H Lee JH Lloyd J Walter P Rane SG. Beneficial metabolic effects of a probiotic via butyrate-induced GLP-1 hormone secretion. J Biol Chem (2013) 288:25088–97. 10.1074/jbc.M113.452516 23836895 PMC3757173

[50] Schroeder FA Lin CL Crusio WE Akbarian S. Antidepressant-like effects of the histone deacetylase inhibitor, sodium butyrate, in the mouse. Biol Psychiatry (2007) 62:55–64. 10.1016/j.biopsych.2006.06.036 16945350

[51] El Aidy S Stilling R Dinan TG Cryan JF Chapter 15: microbiome to brain: unravelling the multidirectional axes of communication, microbial endocrinology: interkingdom signaling in infectious disease and health. Adv Exp Med Biol (2016) 874:301–36. 10.1007/978-3-319-20215-0_15 26589226

[52] Klein C Mathis C Leva G Patte-Mensah C Cassel JC Maitre M y-Hydroxybutyrate (Xyrem) ameliorates clinical symptoms and neuropathology in a mouse model of Alzheimer’s disease. Neurobiol Aging (2015) 36:832–44. 10.1016/j.neurobiolaging.2014.10.003 25457559

[53] Hooper LV Wong MH Thelin A Hansson L Falk PG Gordon JI. Molecular analysis of commensal host-microbial relationships in the intestine. Science (2001) 291:881–4. 10.1126/science.291.5505.881 11157169

[54] Cryan JF Dinan TG. Mind-altering microorganisms: the impact of the gut microbiota on brain and behaviour. Nat Rev Neurosci (2012) 13:701–12. 10.1038/nrn3346 22968153

[55] Wall R Cryan JF Ross RP Fitzgerald GF Dinan TG Stanton C Chapter 10: bacterial neuroactive compounds produced by psychobiotics, microbial endocrinology: the microbiota-gut-brain axis in health and disease. Adv Exp Med Biol (2014) 817:221–39. 10.1007/978-1-4939-0897-4_10 24997036

[56] Claus SP Ellero SL Berger B Krause L Bruttin A Molina J Colonization-induced host-gut microbial metabolic interaction. mBio (2011) 2(2):e00271–10. 10.1128/mBio.00271-10 21363910 PMC3045766

[57] Rhee SH Pothoulakis C Mayer EA. Principles and clinical implications of the brain-gut-enteric microbiota axis. Nat Rev Gastroenterol Hepatol (2009) 6:306–14. 10.1038/nrgastro.2009.35 19404271 PMC3817714

[58] Mayer EA. Gut feelings: the emerging biology of gut-brain communication. Nat Rev Neurosci (2011) 12:453–66. 10.1038/nrn3071 21750565 PMC3845678

[59] Carabotti M Scirocco A Maselli MA Severi C. The gut-brain axis: interactions between enteric microbiota, central and enteric nervous systems. Ann Gastroenterol (2015) 28(2):203–9. 25830558 PMC4367209

[60] Tuohy KM Rouzaud GCM Brück WM Gibson GR Modulation of the human gut microflora towards improved health using prebiotics – assessment of efficacy. Curr Pharm Des (2005) 11:75–90. 10.2174/1381612053382331 15638753

[61] Manning TS Gibson GR Prebiotics. Best Pract Res Clin Gastroenterol (2004) 18:287–98. 10.1016/j.bpg.2003.10.008 15123070

[62] Cummings JH Roberfroid MB Andersson H Barth C Ferro-Luzzi A Ghoos Y A new look at dietary carbohydrate: chemistry, physiology and health. Eur J Clin Nutr (1997) 64:334 10.1038/ejcn.2010.10 9234022

[63] Roberfroid MB. Introducing inulin-type fructans. Br J Nutr (2005) 93:S13. 10.1079/BJN20041350 15877886

[64] Finegold SM Li Z Summanen PH Downes J Thames G Corbett K Xylooligosaccharide increases bifidobacteria but not lactobacilli in human gut microbiota. Food Funct (2014) 5:436–45. 10.1039/c3fo60348b 24513849

[65] Barrett JS Gibson PR. Fermentable oligosaccharides, disaccharides, monosaccharides and polyols (FODMAPs) and nonallergic food intolerance: FODMAPs or food chemicals? Therap Adv Gastroenterol (2012) 5:261–8. 10.1177/1756283X11436241 22778791 PMC3388522

[66] Kootte RS Vrieze A Holleman F Dallinga-Thie GM Zoetendal EG de Vos WM The therapeutic potential of manipulating gut microbiota in obesity and type 2 diabetes mellitus. Diabetes Obes Metab (2012) 14:112–20. 10.1111/j.1463-1326.2011.01483.x 21812894

[67] Witjes JJ van Raalte DH Nieuwdorp M. About the gut microbiome as a pharmacological target in atherosclerosis. Eur J Pharmacol (2015) 763:1–4. 10.1016/j.ejphar.2015.06.023 26096558

[68] Miele L Giorgio V Alberelli MA De Candia E Gasbarrini A Grieco A. Impact of gut microbiota on obesity, diabetes, and cardiovascular disease risk. Curr Cardiol Rep (2015) 17(12):120. 10.1007/s11886-015-0671-z 26497040

[69] Marteau P Seksik P. Tolerance of probiotics and prebiotics. J Clin Gastroenterol (2004) 38:S67–9. 10.1097/01.mcg.0000128929.37156.a7 15220662

[70] Marteau P Flourié B. Tolerance to low-digestible carbohydrates: symptomatology and methods. Br J Nutr (2001) 85:S17. 10.1079/BJN2000258 11321024

[71] Sekirov I Russell SL Antunes LC Finlay BB. Gut microbiota in health and disease. Physiol Rev (2010) 90:859–904. 10.1152/physrev.00045.2009 20664075

[72] Karlsson F Tremaroli V Nielsen J Bäckhed F Assessing the human gut microbiota in metabolic diseases. Diabetes (2013) 62:3341–9. 10.2337/db13-0844 24065795 PMC3781439

[73] Beards E Tuohy K Gibson G. A human volunteer study to assess the impact of confectionery sweeteners on the gut microbiota composition. Br J Nutr (2010) 104:701–8. 10.1017/S0007114510001078 20370946

[74] Inoguchi S Ohashi Y Narai-Kanayama A Aso K Nakagaki T Fujisawa T. Effects of non-fermented and fermented soybean milk intake on faecal microbiota and faecal metabolites in humans. Int J Food Sci Nutr (2012) 63:402–10. 10.3109/09637486.2011.630992 22040525

[75] Vulevic J Juric A Walton GE Claus SP Tzortzis G Toward RE Influence of galacto-oligosaccharide mixture (B-GOS) on gut microbiota, immune parameters and metabonomics in elderly persons. Br J Nutr (2015) 114:586–95. 10.1017/S0007114515001889 26218845

[76] Walton GE Lu C Trogh I Arnaut F Gibson GR. A randomised, double-blind, placebo controlled cross-over study to determine the gastrointestinal effects of consumption of arabinoxylan-oligosaccharides enriched bread in healthy volunteers. Nutr J (2012) 11:36. 10.1186/1475-2891-11-36 22657950 PMC3487838

[77] Cloetens L Broekaert WF Delaedt Y Ollevier F Courtin CM Delcour JA Tolerance of arabinoxylan-oligosaccharides and their prebiotic activity in healthy subjects: a randomised, placebo-controlled cross-over study. Br J Nutr (2010) 103:703–13. 10.1017/S0007114509992248 20003568

[78] Walton GE van den Heuvel EG Kosters MH Rastall RA Tuohy KM Gibson GR. A randomised crossover study investigating the effects of galacto-oligosaccharides on the faecal microbiota in men and women over 50 years of age. Br J Nutr (2012) 107:1466–75. 10.1017/S0007114511004697 21910949

[79] Lecerf JM Dépeint F Clerc E Dugenet Y Niamba CN Rhazi L Xylo-oligosaccharide (XOS) in combination with inulin modulates both the intestinal environment and immune status in healthy subjects, while XOS alone only shows prebiotic properties. Br J Nutr (2012) 108:1847–58. 10.1017/S0007114511007252 22264499

[80] Mitsou EK Turunen K Anapliotis P Zisi D Spiliotis V Kyriacou A. Impact of a jelly containing short-chain fructo-oligosaccharides and *Sideritis euboea* extract on human faecal microbiota. Int J Food Microbiol (2009) 135:112–7. 10.1016/j.ijfoodmicro.2009.08.004 19735957

[81] Baer DJ Stote KS Henderson T Paul DR Okuma K Tagami H The metabolizable energy of dietary resistant maltodextrin is variable and alters fecal microbiota composition in adult men. J Nutr (2014) 144:1023–9. 10.3945/jn.113.185298 24744316

[82] Martínez I Kim J Duffy PR Schlegel VL Walter J. Resistant starches types 2 and 4 have differential effects on the composition of the fecal microbiota in human subjects. PLoS One (2010) 5:e15046. 10.1371/journal.pone.0015046 21151493 PMC2993935

[83] Holscher HD Bauer LL Gourineni V Pelkman CL Fahey GC Jr Swanson KS. Agave inulin supplementation affects the fecal microbiota of healthy adults participating in a randomized, double-blind, placebo-controlled, crossover trial. J Nutr (2015) 145:2025–32. 10.3945/jn.115.217331 26203099

[84] Costabile A Kolida S Klinder A Gietl E Bäuerlein M Frohberg C A double-blind, placebo-controlled, cross-over study to establish the bifidogenic effect of a very-long-chain inulin extracted from globe artichoke (*Cynara scolymus*) in healthy human subjects. Br J Nutr (2010) 104:1007–17. 10.1017/S0007114510001571 20591206

[85] Ramirez-Farias C Slezak K Fuller Z Duncan A Holtrop G Louis P. Effect of inulin on the human gut microbiota: stimulation of *Bifidobacterium adolescentis* and *Faecalibacterium prausnitzii*. Br J Nutr (2009) 101:533. 10.1017/S0007114508019880 18590586

[86] Linetzky Waitzberg D Alves Pereira CC Logullo L Manzoni Jacintho T Almeida D Teixeira da Silva ML Microbiota benefits after inulin and partially hydrolized guar gum supplementation: a randomized clinical trial in constipated women. Nutr Hosp (2012) 27:123–9. 10.1590/S0212-16112012000100014 22566311

[87] Holscher HD Caporaso JG Hooda S Brulc JM Fahey GC Jr Swanson KS. Fiber supplementation influences phylogenetic structure and functional capacity of the human intestinal microbiome: follow-up of a randomized controlled trial. Am J Clin Nutr (2015) 101:55–64. 10.3945/ajcn.114.092064 25527750

[88] Hooda S Boler BM Serao MC Brulc JM Staeger MA Boileau TW 454 pyrosequencing reveals a shift in fecal microbiota of healthy adult men consuming polydextrose or soluble corn fiber. J Nutr (2012) 142:1259–65. 10.3945/jn.112.158766 22649263

[89] Whisner CM Martin BR Nakatsu CH Story JA MacDonald-Clarke CJ McCabe LD Soluble corn fiber increases calcium absorption associated with shifts in the gut microbiome: a randomized dose-response trial in free-living pubertal females. J Nutr (2016) 146:1298–306. 10.3945/jn.115.227256 27281813

[90] Yang J Summanen PH Henning SM Hsu M Lam H Huang J Xylooligosaccharide supplementation alters gut bacteria in both healthy and prediabetic adults : a pilot study. Front Physiol (2015) 6:216. 10.3389/fphys.2015.00216 26300782 PMC4528259

[91] Li T Lu X Yang X Evaluation of clinical safety and beneficial effects of stachyose-enriched a-galacto-oligosaccharides on gut microbiota and bowel function in humans. Food Funct (2017) 8:262–9. 10.1039/C6FO01290F 28001151

[92] Tai N Wong FS Wen L The role of gut microbiota in the development of type 1, type 2 diabetes mellitus and obesity. Rev Endocr Metab Disord (2015) 16:55–65. 10.1007/s11154-015-9309-0 25619480 PMC4348024

[93] Ley RE Turnbaugh PJ Klein S Gordon JI. Microbial ecology: human gut microbes associated with obesity. Nature (2006) 444:1022–3. 10.1038/4441022a 17183309

[94] Zhang H DiBaise JK Zuccolo A Kudrna D Braidotti M Yu Y Human gut microbiota in obesity and after gastric bypass. Proc Natl Acad Sci U S A (2009) 106:2365–70. 10.1073/pnas.0812600106 19164560 PMC2629490

[95] Ley RE Bäckhed F Turnbaugh P Lozupone CA Knight RD Gordon JI. Obesity alters gut microbial ecology. Proc Natl Acad Sci U S A (2005) 102:11070–5. 10.1073/pnas.0504978102 16033867 PMC1176910

[96] Armougom F Henry M Vialettes B Raccah D Raoult D. Monitoring bacterial community of human gut microbiota reveals an increase in *Lactobacillus* in obese patients and methanogens in anorexic patients. PLoS One (2009) 4:e7125. 10.1371/journal.pone.0007125 19774074 PMC2742902

[97] Turnbaugh PJ Hamady M Yatsunenko T Cantarel BL Duncan A Ley RE A core gut microbiome in obese and lean twins. Nature (2009) 457:480–4. 10.1038/nature07540 19043404 PMC2677729

[98] Furet JP Kong LC Tap J Poitou C Basdevant A Bouillot JL Differential adaptation of human gut microbiota to bariatric surgery-induced weight loss: links with metabolic and low-grade inflammation markers. Diabetes (2010) 59:3049–57. 10.2337/db10-0253 20876719 PMC2992765

[99] Schwiertz A Taras D Schäfer K Beijer S Bos NA Donus C Microbiota and SCFA in lean and overweight healthy subjects. Obesity (2010) 18:190–5. 10.1038/oby.2009.167 19498350

[100] Duncan SH Lobley GE Holtrop G Ince J Johnstone AM Louis P Human colonic microbiota associated with diet, obesity and weight loss. Int J Obes (Lond) (2008) 32:1720–4. 10.1038/ijo.2008.155 18779823

[101] Collado MC Isolauri E Laitinen K Salminen S. Distinct composition of gut microbiota during pregnancy in overweight and normal-weight women. Am J Clin Nutr (2008) 88(4):894–9. 18842773 10.1093/ajcn/88.4.894

[102] Beilby J Definition of metabolic syndrome: report of the National Heart, Lung, and Blood Institute/American Heart Association Conference on scientific issues related to definition. Clin Biochem Rev (2004) 109:433–8. 10.1161/01.CIR.0000111245.75752.C6 14744958

[103] Egshatyan L Kashtanova D Popenko A Tkacheva O Tyakht A Alexeev D Gut microbiota and diet in patients with different glucose tolerance. Endocr Connect (2016) 5:1–9. 10.1530/EC-15-0094 26555712 PMC4674628

[104] Le Chatelier E Nielsen T Qin J Prifti E Hildebrand F Falony G Richness of human gut microbiome correlates with metabolic markers. Nature (2013) 500:541–6. 10.1038/nature12506 23985870

[105] Karlsson FH Tremaroli V Nookaew I Bergström G Behre CJ Fagerberg B Gut metagenome in European women with normal, impaired and diabetic glucose control. Nature (2013) 498:99–103. 10.1038/nature12198 23719380

[106] Qin J Li Y Cai Z Li S Zhu J Zhang F A metagenome-wide association study of gut microbiota in type 2 diabetes. Nature (2012) 490:55–60. 10.1038/nature11450 23023125

[107] Libby P. Inflammation in atherosclerosis. Nature (2002) 420:868–74. 10.1038/nature01323 12490960

[108] Minihane AM Vinoy S Russell WR Baka A Roche HM Tuohy KM Low-grade inflammation, diet composition and health: current research evidence and its translation. Br J Nutr (2015) 114:999–1012. 10.1017/S0007114515002093 26228057 PMC4579563

[109] Hotamisligil GS Inflammation and metabolic disorders. Nature (2006) 444:860–7. 10.1038/nature05485 17167474

[110] Cooke AA Connaughton RM Lyons CL McMorrow AM Roche HM. Fatty acids and chronic low grade inflammation associated with obesity and the metabolic syndrome. Eur J Pharmacol (2016) 785:207–14. 10.1016/j.ejphar.2016.04.021 27083551

[111] Nagpal R Kumar M Yadav AK Hemalatha R Yadav H Marotta F Gut microbiota in health and disease: an overview focused on metabolic inflammation. Benef Microbes (2016) 7(2):181–94. 10.3920/bm2015.0062 26645350

[112] Andoh A Tsuijikawa T Sasaki M Mitsuyama K Suzuki Y Matsui T Faecal microbiota profile of Crohn’s disease determined by terminal restriction fragment length polymorphism analysis. Aliment Pharmacol Ther (2009) 29:75–82. 10.1111/j.1365-2036.2008.03860.x 18945264

[113] Swidsinski A Weber J Loening-Baucke V Hale LP Lochs H. Spatial organization and composition of the mucosal flora in patients with inflammatory bowel disease. J Clin Microbiol (2005) 43:3380–9. 10.1128/JCM.43.7.3380-3389.2005 16000463 PMC1169142

[114] Fitzmorris PS Colantonio LD Torrazza Perez E Smith I Debajyoti Kakati D Aziz Malik T Impact of metabolic syndrome on the hospitalization rate of Crohn’s disease patients seen at a tertiary care center: a retrospective cohort study. Digestion (2015) 91:257–62. 10.1159/000380763 25833139

[115] Yorulmaz E Adali G Yorulmaz H Ulasoglu C Tasan G Tuncer I. Metabolic syndrome frequency in inflammatory bowel diseases. Saudi J Gastroenterol (2011) 17:376–82. 10.4103/1319-3767.87177 22064334 PMC3221110

[116] Larsen N Vogensen FK van den Berg FW Nielsen DS Andreasen AS Pedersen BK Gut microbiota in human adults with type 2 diabetes differs from non-diabetic adults. PLoS One (2010) 5:e9085. 10.1371/journal.pone.0009085 20140211 PMC2816710

[117] Karlsson FH Fåk F Nookaew I Tremaroli V Fagerberg B Petranovic D Symptomatic atherosclerosis is associated with an altered gut metagenome. Nat Commun (2012) 3:1245. 10.1038/ncomms2266 23212374 PMC3538954

[118] El Aidy S Derrien M Merrifield CA Levenez F Doré J Boekschoten MV Gut bacteria-host metabolic interplay during conventionalisation of the mouse germfree colon. ISME J (2013) 7:743–55. 10.1038/ismej.2012.142 23178667 PMC3603396

[119] Teixeira TFS Grzeskowiak LM Salminen S Laitinen K Bressan J Carmo Gouveia Peluzio M Faecal levels of *Bifidobacterium* and *Clostridium coccoides* but not plasma lipopolysaccharide are inversely related to insulin and HOMA index in women. Clin Nutr (2013) 32:1017–22. 10.1016/j.clnu.2013.02.008 23538004

[120] Sato J Kanazawa A Ikeda F Yoshihara T Goto H Abe H Gut dysbiosis and detection of ‘Live gut bacteria’ in blood of Japanese patients with type 2 diabetes. Diabetes Care (2014) 37:2343–50. 10.2337/dc13-2817 24824547

[121] Nadal I Santacruz A Marcos A Warnberg J Garagorri JM Moreno LA Shifts in clostridia, *Bacteroides* and immunoglobulin-coating fecal bacteria associated with weight loss in obese adolescents. Int J Obes (Lond) (2009) 33:758–67. 10.1038/ijo.2008.260 19050675

[122] Santacruz A Marcos A Wärnberg J Martí A Martin-Matillas M Campoy C Interplay between weight loss and gut microbiota composition in overweight adolescents. Obesity (2009) 17:1906–15. 10.1038/oby.2009.112 19390523

[123] Ananthakrishnan AN McGinley EL Binion DG. Excess hospitalisation burden associated with *Clostridium difficile* in patients with inflammatory bowel disease. Gut (2008) 57:205–10. 10.1136/gut.2007.128231 17905821

[124] Gagnière J Raisch J Veziant J Barnich N Bonnet R Buc E Gut microbiota imbalance and colorectal cancer. World J Gastroenterol (2016) 22:501–18. 10.3748/wjg.v22.i2.501 26811603 PMC4716055

[125] Stackebrandt E Kramer I Swiderski J Hippe H. Phylogenetic basis for a taxonomic dissection of the genus *Clostridium*. FEMS Immunol Med Microbiol (1999) 24:253–8. 10.1111/j.1574-695X.1999.tb01291.x 10397308

[126] Petriz BA Castro AP Almeida JA Gomes CP Fernandes GR Kruger RH Exercise induction of gut microbiota modifications in obese, non-obese and hypertensive rats. BMC Genomics (2014) 15:511. 10.1186/1471-2164-15-511 24952588 PMC4082611

[127] Ignacio A Fernandes MR Rodrigues VA Groppo FC Cardoso AL Avila-Campos MJ Correlation between body mass index and faecal microbiota from children. Clin Microbiol Infect (2016) 22(3):258.e1–8. 10.1016/j.cmi.2015.10.031 26551842

[128] Ejtahed HS Mohtadi-Nia J Homayouni-Rad A Niafar M Asghari-Jafarabadi M Mofid V. Probiotic yogurt improves antioxidant status in type 2 diabetic patients. Nutrition (2012) 28:539–43. 10.1016/j.nut.2011.08.013 22129852

[129] Sanchez M Drapeau V Emady-Azar S Lepage M Rezzonico E Ngom-Bru C Effect of *Lactobacillus rhamnosus* CGMCC1.3724 supplementation on weight loss and maintenance in obese men and women. Br J Nutr (2014) 111:1507–19. 10.1017/S0007114513003875 24299712

[130] Kalliomäki M Collado MC Salminen S Isolauri E. Early differences in fecal microbiota composition in children may predict overweight. Am J Clin Nutr (2008) 87(3):534–8. 18326589 10.1093/ajcn/87.3.534

[131] Santacruz A Collado MC García-Valdés L Segura MT Martín-Lagos JA Anjos T Gut microbiota composition is associated with body weight, weight gain and biochemical parameters in pregnant women. Br J Nutr (2010) 104:83–92. 10.1017/S0007114510000176 20205964

[132] Xu X Hui H Cai D. [Differences in fecal *Bifidobacterium* species between patients with type 2 diabetes and healthy individuals]. Nan Fang Yi Ke Da Xue Xue Bao (2012) 32(4):531–3. 22543136

[133] Cani PD Neyrinck AM Fava F Knauf C Burcelin RG Tuohy KM Selective increases of bifidobacteria in gut microflora improve high-fat-diet-induced diabetes in mice through a mechanism associated with endotoxaemia. Diabetologia (2007) 50:2374–83. 10.1007/s00125-007-0791-0 17823788

[134] Cani PD Amar J Iglesias MA Poggi M Knauf C Bastelica D Metabolic endotoxemia initiates obesity and insulin resistance. Diabetes (2007) 56:1761–72. 10.2337/db06-1491 17456850

[135] Cani PD Possemiers S Van de Wiele T Guiot Y Everard A Rottier O Changes in gut microbiota control inflammation in obese mice through a mechanism involving GLP-2-driven improvement of gut permeability. Gut (2009) 58:1091–103. 10.1136/gut.2008.165886 19240062 PMC2702831

[136] Griffiths EA Duffy LC Schanbacher FL Qiao H Dryja D Leavens A In vivo effects of bifidobacteria and lactoferrin on gut endotoxin concentration and mucosal immunity in Balb/c mice. Dig Dis Sci (2004) 49:579–89. 10.1023/B:DDAS.0000026302.92898.ae 15185861

[137] Wang Z Xiao G Yao Y Guo S Lu K Sheng Z. The role of bifidobacteria in gut barrier function after thermal injury in rats. J Trauma (2006) 61:650–7. 10.1097/01.ta.0000196574.70614.27 16967002

[138] El Aidy S Derrien M Aardema R Hooiveld G Richards SE Dane A Transient inflammatory-like state and microbial dysbiosis are pivotal in establishment of mucosal homeostasis during colonisation of germ-free mice. Benef Microbes (2014) 5:67–77. 10.3920/BM2013.0018 24322881

[139] Erridge C. Diet, commensals and the intestine as sources of pathogen-associated molecular patterns in atherosclerosis, type 2 diabetes and non-alcoholic fatty liver disease. Atherosclerosis (2011) 216:1–6. 10.1016/j.atherosclerosis.2011.02.043 21439567

[140] Spreadbury I. Comparison with ancestral diets suggests dense acellular carbohydrates promote an inflammatory microbiota, and may be the primary dietary cause of leptin resistance and obesity. Diabetes Metab Syndr Obes (2012) 5:175–89. 10.2147/DMSO.S33473 22826636 PMC3402009

[141] Tonucci LB Olbrich Dos Santos KM Licursi de Oliveira L Rocha Ribeiro SM Duarte Martino HS Effects of probiotics on glycemic control and inflammation in type 2 diabetes mellitus: a randomized, double-blind, placebocontrolled study. Clin Nutr (2017) 36(1):85–92. 10.1016/j.clnu.2015.11.011 26732026

[142] Moroti C Souza Magri LF de Rezende Costa M Cavallini DC Sivieri K. Effect of the consumption of a new symbiotic shake on glycemia and cholesterol levels in elderly people with type 2 diabetes mellitus. Lipids Health Dis (2012) 11:29. 10.1186/1476-511X-11-29 22356933 PMC3305430

[143] Sepp E Kolk H Lõivukene K Mikelsaar M. Higher blood glucose level associated with body mass index and gut microbiota in elderly people. Microb Ecol Health Dis (2014) 25:22857. 10.3402/mehd.v25.22857 24936169 PMC4048595

[144] Sepp E Lõivukene K Julge K Voor T Mikelsaar M. The association of gut microbiota with body weight and body mass index in preschool children of Estonia. Microb Ecol Health Dis (2013) 24:19231. 10.3402/mehd.v24i0.19231 24009544 PMC3758928

[145] Brugger SD Baumberger C Jost M Jenni W Brugger U Mühlemann K. Automated counting of bacterial colony forming units on agar plates. PLoS One (2012) 7:e33695. 10.1371/journal.pone.0033695 22448267 PMC3308999

[146] Maccaferri S Vitali B Klinder A Kolida S Ndagijimana M Laghi L Rifaximin modulates the colonic microbiota of patients with Crohn’s disease: an in vitro approach using a continuous culture colonic model system. J Antimicrob Chemother (2010) 65:2556–65. 10.1093/jac/dkq345 20852272

[147] Khachatryan ZA Ktsoyan ZA Manukyan GP Kelly D Ghazaryan KA Aminov RI. Predominant role of host genetics in controlling the composition of gut microbiota. PLoS One (2008) 3:e3064. 10.1371/journal.pone.0003064 18725973 PMC2516932

[148] Vulevic J Juric A Tzortzis G Gibson GR. A mixture of trans-galactooligosaccharides reduces markers of metabolic syndrome and modulates the fecal microbiota and immune function of overweight adults 1–3. J Nutr (2013) 143:324–31. 10.3945/jn.112.166132 23303873

[149] Sawin EA De Wolfe TJ Aktas B Stroup BM Murali SG Steele JL Glycomacropeptide is a prebiotic that reduces *Desulfovibrio* bacteria, increases cecal short-chain fatty acids, and is anti-inflammatory in mice. Am J Physiol Gastrointest Liver Physiol (2015) 309:G590–601. 10.1152/ajpgi.00211.2015 26251473 PMC4593820

[150] Rowan F Docherty NG Murphy M Murphy B Calvin Coffey J O’Connell PR. *Desulfovibrio* bacterial species are increased in ulcerative colitis. Dis Colon Rectum (2010) 53:1530–6. 10.1007/DCR.0b013e3181f1e620 20940602

[151] Karlsson CL Onnerfält J Xu J Molin G Ahrné S Thorngren-Jerneck K. The microbiota of the gut in preschool children with normal and excessive body weight. Obesity (Silver Spring) (2012) 20:2257–61. 10.1038/oby.2012.110 22546742

[152] Mitchell CM Davy BM Halliday TM Hulver MW Neilson AP Ponder MA The effect of prebiotic supplementation with inulin on cardiometabolic health: rationale, design, and methods of a controlled feeding efficacy trial in adults at risk of type 2 diabetes. Contemp Clin Trials (2015) 45:328–37. 10.1016/j.cct.2015.10.012 26520413 PMC4743874

[153] Rault-Nania MH Demougeot C Gueux E Berthelot A Dzimira S Rayssiguier Y Inulin supplementation prevents high fructose diet-induced hypertension in rats. Clin Nutr (2008) 27:276–82. 10.1016/j.clnu.2008.01.015 18358572

[154] Arora T Loo RL Anastasovska J Gibson GR Tuohy KM Sharma RK Differential effects of two fermentable carbohydrates on central appetite regulation and body composition. PLoS One (2012) 7:e43263. 10.1371/journal.pone.0043263 22952656 PMC3430697

[155] Weitkunat K Schumann S Petzke KJ Blaut M Loh G Klaus S. Effects of dietary inulin on bacterial growth, short-chain fatty acid production and hepatic lipid metabolism in gnotobiotic mice. J Nutr Biochem (2015) 26:929–37. 10.1016/j.jnutbio.2015.03.010 26033744

[156] Salazar N Dewulf EM Neyrinck AM Bindels LB Cani PD Mahillon J Inulin-type fructans modulate intestinal *Bifidobacterium* species populations and decrease fecal short-chain fatty acids in obese women. Clin Nutr (2015) 34:501–7. 10.1016/j.clnu.2014.06.001 24969566

[157] Bruhwyler J Carreer F Demanet E Jacobs H. Digestive tolerance of inulin-type fructans: a double-blind, placebo-controlled, cross-over, dose-ranging, randomized study in healthy volunteers. Int J Food Sci Nutr (2009) 60:165–75. 10.1080/09637480701625697 18608562

[158] Housez B Cazaubiel M Vergara C Bard JM Adam A Einerhand A Evaluation of digestive tolerance of a soluble corn fibre. J Hum Nutr Diet (2012) 25:488–96. 10.1111/j.1365-277X.2012.01252.x 22672058

[159] Myers MG Jr Leibel RL Seeley RJ Schwartz MW. Obesity and leptin resistance: distinguishing cause from effect. Trends Endocrinol Metab (2010) 21:643–51. 10.1016/j.tem.2010.08.002 20846876 PMC2967652

[160] Martin SS Qasim A Reilly MP. Leptin resistance: a possible interface of inflammation and metabolism in obesity-related cardiovascular disease. J Am Coll Cardiol (2008) 52:1201–10. 10.1016/j.jacc.2008.05.060 18926322 PMC4556270

[161] DiBaise JK Zhang H Crowell MD Krajmalnik-Brown R Decker GA Rittmann BE. Gut microbiota and its possible relationship with obesity. Mayo Clin Proc (2008) 83:460–9. 10.4065/83.4.460 18380992

[162] Samuel BS Gordon JI. A humanized gnotobiotic mouse model of host-archaeal-bacterial mutualism. Proc Natl Acad Sci U S A (2006) 103:10011–6. 10.1073/pnas.0602187103 16782812 PMC1479766

[163] Neel JV Diabetes mellitus: a ‘thrifty’ genotype rendered detrimental by ‘progress’? 1962. Bull World Health Organ (1999) 77:353–62. PMC2557712 10516792

[164] Prentice AM Rayco-Solon P Moore SE. Insights from the developing world: thrifty genotypes and thrifty phenotypes. Proc Nutr Soc (2005) 64:153–61. 10.1079/PNS2005421 15960860

[165] Kyung Lee Y Mazmanian SK. Has the microbiota played a critical role in the evolution of the adaptive immune system? Science (2010) 330(6012):1768–73. 10.1126/science.1195568 21205662 PMC3159383

[166] Ley RE Lozupone CA Hamady M Knight R Gordon JI Worlds within worlds: evolution of the vertebrate gut microbiota. Nat Rev Microbiol (2008) 6(10):776–88. 10.1038/nrmicro1978 18794915 PMC2664199

[167] Alcock J Maley CC Aktipis CA. Is eating behavior manipulated by the gastrointestinal microbiota? Evolutionary pressures and potential mechanisms. Bioessays (2014) 36:940–9. 10.1002/bies.201400071 25103109 PMC4270213

[168] Perry RJ Peng L Barry NA Cline GW Zhang D Cardone RL Acetate mediates a microbiome-brain–β-cell axis to promote metabolic syndrome. Nature (2016) 534:213–7. 10.1038/nature18309 27279214 PMC4922538

[169] Whelan K. Mechanisms and effectiveness of prebiotics in modifying the gastrointestinal microbiota for the management of digestive disorders. Proc Nutr Soc (2013) 72:288–98. 10.1017/S0029665113001262 23680358

[170] Brown K DeCoffe D Molcan E Gibson DL. Diet-induced dysbiosis of the intestinal microbiota and the effects on immunity and disease. Nutrients (2012) 4:1095–119. 10.3390/nu4081095 23016134 PMC3448089

[171] van Duynhoven J Vaughan EE Jacobs DM Kemperman RA van Velzen EJ Gross G Metabolic fate of polyphenols in the human superorganism. Proc Natl Acad Sci U S A (2011) 108(Suppl):4531–8. 10.1073/pnas.1000098107 20615997 PMC3063601

[172] Dumas ME Barton RH Toye A Cloarec O Blancher C Rothwell A Metabolic profiling reveals a contribution of gut microbiota to fatty liver phenotype in insulin-resistant mice. Proc Natl Acad Sci U S A (2006) 103:12511–6. 10.1073/pnas.0601056103 16895997 PMC1567909

[173] Kitai T Kirsop J Tang WH. Exploring the microbiome in heart failure. Curr Heart Fail Rep (2016) 13(2):103–9. 10.1007/s11897-016-0285-9 26886380 PMC4791185

[174] Wang Z Klipfell E Bennett BJ Koeth R Levison BS Dugar B Gut flora metabolism of phosphatidylcholine promotes cardiovascular disease. Nature (2011) 472:57–63. 10.1038/nature09922 21475195 PMC3086762

[175] Sharon G Garg N Debelius J Knight R Dorrestein PC Mazmanian SK. Specialized metabolites from the microbiome in health and disease. Cell Metab (2014) 20:719–30. 10.1016/j.cmet.2014.10.016 25440054 PMC4337795

[176] Esser N Paquot N Scheen AJ Inflammatory markers and cardiometabolic diseases. Acta Clin Belg (2015) 70:193–9. 10.1179/2295333715Y.0000000004 26103538

[177] Cani PD Possemiers S Van de Wiele T Guiot Y Everard A Rottier O Changes in gut microbiota control inflammation in obese mice through a mechanism involving GLP-2-driven improvement of gut permeability. Gut (2009) 58:1091–103. 10.1136/gut.2008.165886 19240062 PMC2702831

[178] Cani PD Bibiloni R Knauf C Waget A Neyrinck AM Delzenne NM Changes in gut microbiota control metabolic diet–induced obesity and diabetes in mice. Diabetes (2008) 57:1470–81. 10.2337/db07-1403 18305141

[179] Sanmiguel C Gupta A Mayer EA. Gut microbiome and obesity: a plausible explanation for obesity. Curr Obes Rep (2015) 4:250–61. 10.1007/s13679-015-0152-0 26029487 PMC4443745

[180] Hill DR Spence JR Gastrointestinal organoids; understanding the molecular basis of the host-microbe interface. Cell Mol Gastrointerol Hepatol (2017) 3(2):138–49. 10.1016/j.jcmgh.2016.11.007 PMC5331777 28275681

[181] Fatehullah A Tan SH Barker N. Organoids as an in vitro model of human development and disease. Nat Cell Biol (2016) 18(3):246–54. 10.1038/ncb3312 26911908

